# Evaluation of the role of lisdexamfetamine on attention-deficit/hyperactivity disorder common psychiatric comorbidities: mechanistic insights on binge eating disorder and depression

**DOI:** 10.1192/j.eurpsy.2022.737

**Published:** 2022-09-01

**Authors:** J.R. Gutiérrez Casares, J. Quintero, P. Rodríguez, C. Montoto, T. Pozo-Rubio, C. Segú-Vergés, M. Coma

**Affiliations:** 1 Hospital Perpetuo Socorro, Unidad Ambulatoria De Psiquiatría Y Salud Mental De La Infancia, Niñez Y Adolescencia, Badajoz, Spain; 2 Hospital Universitario Infanta Leonor. Universidad Complutense., Servicio De Psiquiatría, Madrid, Spain; 3 Takeda Farmacéutica España, Medical Department, Madrid, Spain; 4 Anaxomics Biotech, Molecular Health Department, Barcelona, Spain; 5 Anaxomics SL, Molecular Physiology, Barcelona, Spain

**Keywords:** Artificial intelligence, lisdexamfetamine, attention-deficit/hyperactivity disorder, Psychiatric comorbidities

## Abstract

**Introduction:**

Attention-deficit/hyperactivity disorder (ADHD) is a psychiatric condition in which children suffer from inattentiveness, hyperactivity, and or impulsivity. ADHD patients frequently present comorbid psychiatric disorders: in adults, the most common are depression, substance-related disorders, anxiety, and eating disorders. Children and adolescents present conduct disorders, learning disorders, anxiety and depression. Since ADHD and its psychiatric comorbidities share similarities, a partial overlap of their pathophysiological mechanisms has been suggested. ADHD, can be treated with lisdexamfetamine (LDX), a prodrug indicated by the FDA as treatment for binge eating disorder (BED) and ADHD.

**Objectives:**

To evaluate, through a systems biology-based *in silico* method, the efficacy of LDX as first-line ADHD treatment to improve ADHD psychiatric comorbidities. Furthermore, we explored the molecular mechanisms behind LDX’s action.

**Methods:**

We used the systems biology- and artificial intelligence-based Therapeutic Performance Mapping System (TPMS) technology to characterise and model ADHD comorbidities. Artificial neural networks (ANNs) algorithms were used to identify specific relationships between protein sets. Finally, we modelled the mechanisms of LDX for the most relevant comorbidities by using sampling methods and comorbidity-specific virtual patients in each case.

**Results:**

This study predicts a strong relationship between LDX’s targets and proteins involved in BED and depression (Fig 1). Our results could be explained not only by LDX role in neurotransmitter regulation, but also by modulation of neuroplasticity (BDNF/NTRK2, GSK3), neuroinflammation (interleukins, inflammasome), oxidative stress (NOS2, SOD), and the hypothalamic-pituitary-adrenal (HPA) axis (CRH, CRHR1).

**Figure:**
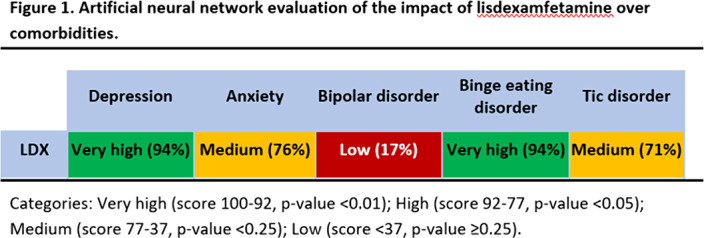

**Conclusions:**

These findings could be used in pre-clinical and clinical future investigations to assess optimal treatment for ADHD patients with psychiatric comorbidities.

**Disclosure:**

JRGC: speaker for Takeda and Shire, research funding from Shire and Lumbeck, collaborations with Laboratoires Servier JQ: speaker or scientific advisor for Takeda, Janssen, Rubio. Investigation funding: Instituto de Salud Carlos III. PR, CM, TPR: full-ti

